# Error Modeling and Calibration for Encoded Sun Sensors

**DOI:** 10.3390/s130303217

**Published:** 2013-03-07

**Authors:** Qiaoyun Fan, Guangjun Zhang, Jian Li, Xinguo Wei, Xiaoyang Li

**Affiliations:** School of Instrumentation Science and Opto-electronics Engineering, Beijing University of Aeronautics and Astronautics, No. 37 Xueyuan Road, Haidian District, Beijing 100191, China; E-Mails: fqy2003@aspe.buaa.edu.cn (Q.F.); lijian_0355@163.com (J.L.); wxg@buaa.edu.cn (X.W.); lxy890123@tju.edu.cn (X.L.)

**Keywords:** encoded sun sensor, error modeling, calibration, fine code

## Abstract

Error factors in the encoded sun sensor (ESS) are analyzed and simulated. Based on the analysis results, an ESS error compensation model containing structural errors and fine-code algorithm errors is established, and the corresponding calibration method for model parameters is proposed. As external parameters, installation deviation between ESS and calibration equipment are introduced to the ESS calibration model, so that the model parameters can be calibrated accurately. The experimental results show that within plus/minus 60 degree of incident angle, the ESS measurement accuracy after compensation is three times higher on average than that before compensation.

## 1. Introduction

The encoded sun sensor (ESS), an important attitude measurement component in the satellite attitude control system [1,2], is at present successfully applied in ninety percent of Chinese satellites for its simple structure and proven technology. However, its accuracy is limited by manufacturing tolerances of the components, assembly deviations and algorithm approximation errors in signal processing, *etc.* If an effective model cannot be found to compensate these errors, the measurement accuracy of a traditional ESS will not meet the increased demands of the spacecraft [3], so in this paper, error factors which reduce the accuracy of encoded sun sensors are analyzed. Accordingly an error compensation model for an encoded sun sensor is established, and an accurate calibration method for model parameters is also proposed.

## 2. Structure and Working Principle of ESS

**Figure 1.**
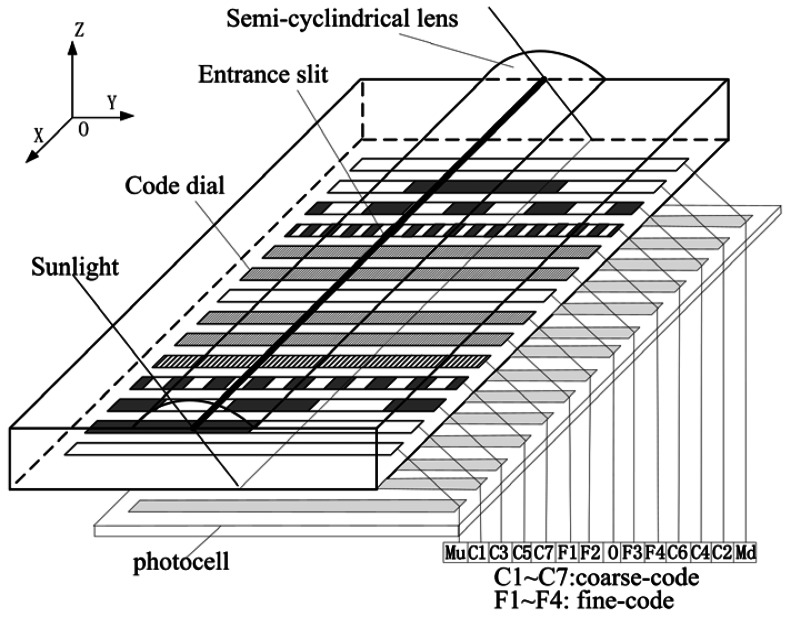
Optical sensing unit of an encoder sun sensor.

An encoded sun sensor is mainly made up of an optical sensing unit and signal processing circuits. The optical sensing unit usually includes optical components (such as a semi-cylindrical lens) with etched entrance slit, code dial and embedded photocell, as shown in Figure 1. Its working principle is: Sunlight is projected onto the code dial through entrance slit in different incident angles. There is a series of encoded rows on the code dial. Each row is etched with an opaque-transparent alternating grid. Photocells beneath each encoded row receive sunlight through the code dial and convert them into a current signal which is related with the incident angle of sunlight. These current signals are further processed by signal processing circuit into an angle output.

## 3. Error Analysis of ESS

Each step of the imaging and signal processing has been investigated to find the possible error factors [4–6]. Simulation for two types of product has been done to estimate the degree of influence of the various error factors.

### 3.1. Distance Deviation between Entrance Slit and Code Dial

**Figure 2.**
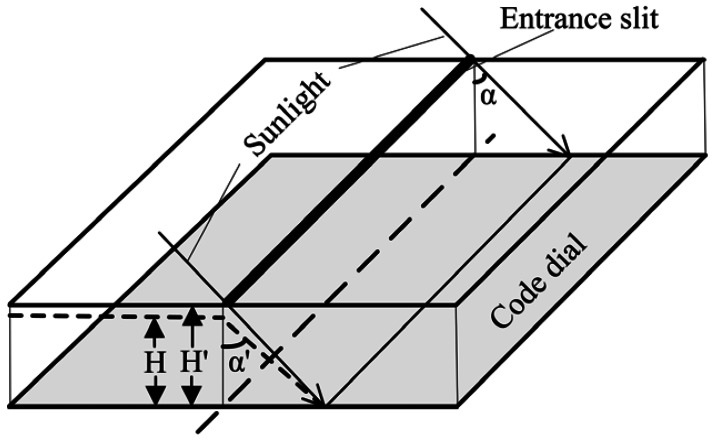
Deviation of distance between entrance slit and code dial.

The encoded rows on code dial are etched according to the rule of *y* = *H* tan(*α*), where H is the distance between entrance slit and code dial. The ideal value of the distance is *H*, but there is always a deviation *ΔH* between ideal value and actual value due to limited manufacturing accuracy. Let *y* be the position of sunlight on the code dial when incident angle (*i.e*., measurement angle) is *α*. The output angle of ESS can be expressed as *α*′ = atan(*y*/*H*), while the actual incident angle of sunlight is *α* = atan(*y*/*H*′), which cause error as shown in Figure 2.

**Figure 3.**
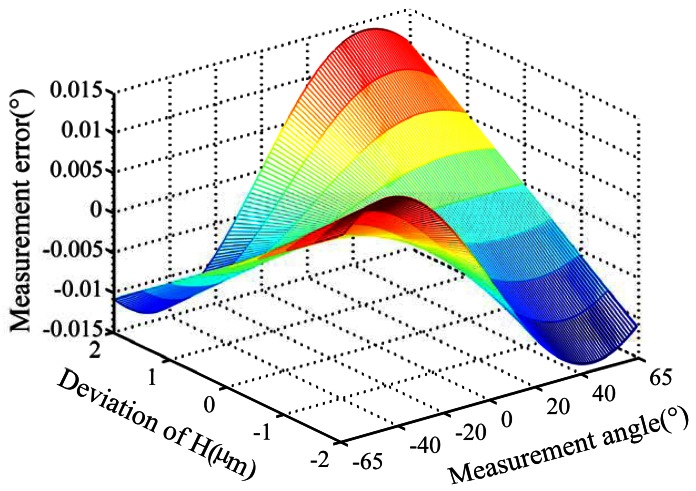
Measurement error caused by distance deviation *ΔH.*

Measurement errors caused by distance deviation *ΔH* for two types of product (designated as A and B) were analyzed by simulation. The distance *H* is 4.124 mm for product A and 6.584 mm for product B. The simulation is done under the following conditione: distance deviation *ΔH* is ±2 microns, and the range of measurement angle is (−62°∼62°). Figure 3 shows the simulation result for product A.

Simulated results are as follows:

(1) Measurement errors caused by distance deviation *ΔH* will increase when the distance between the entrance slit and code dial decreases or the incident angle increases.
(2) For Product A, measurement error will exceed 0.015° when the distance deviation exceeds two micron, as shown in Figure 3. For Product B, measurement error will exceed 0.008° when the distance deviation exceeds two microns. It can be seen that the distance deviation *ΔH* will lead eventually to considerable measurement errors, so the distance deviation *ΔH* cannot be ignored.

### 3.2. Misalignment between Entrance Slit and Center Line of Code Dial

**Figure 4.**
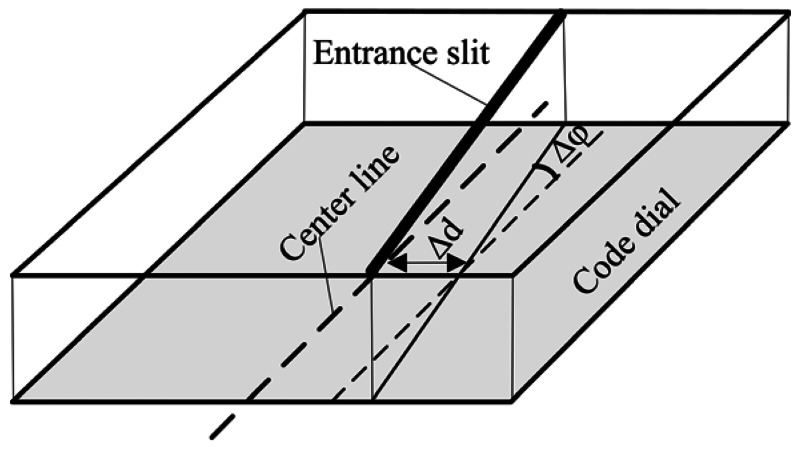
Misalignment between entrance slit and code dial.

In an ideal condition, the entrance slit should be aligned with center line of the code dial, but due to assembly deviation between the semi-cylindrical lens and code dial, offset *Δd* and tilt *Δφ* between entrance slit and center line of code dial will inevitably occur, which cause errors as shown in Figure 4.

**Figure 5.**
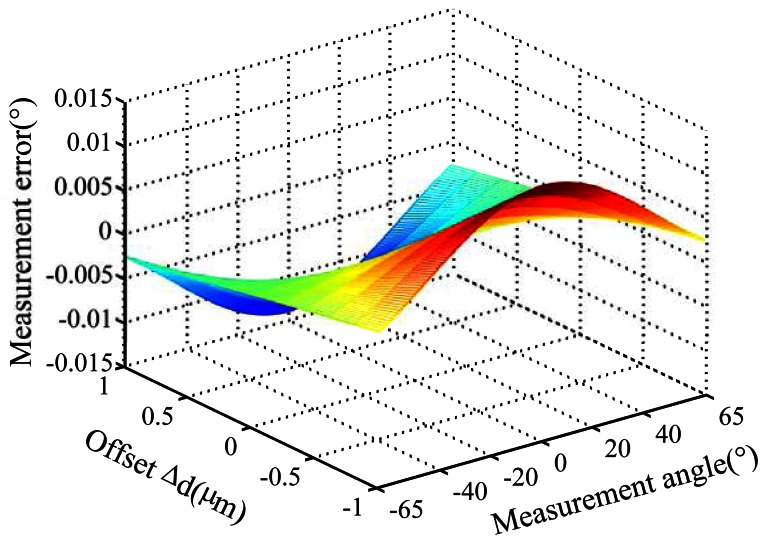
Measurement error caused by offset deviation.

**Figure 6.**
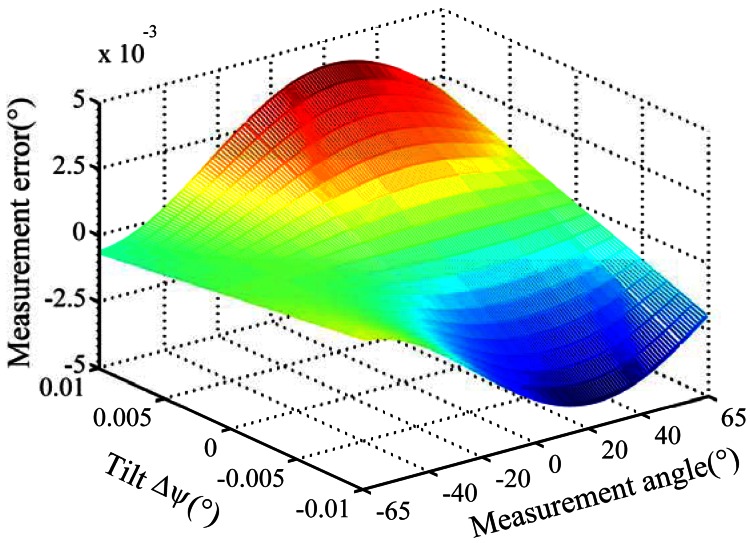
Measurement error caused by tilt deviation.

**Figure 7.**
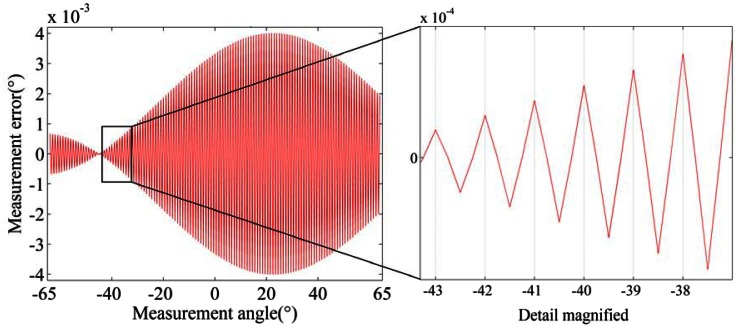
Measurement error caused by tilt deviation when simulated at 0.5° intervals.

Measurement errors caused by offset (*Δd*) and tilt (*Δφ*) deviation for product A and B are analyzed by simulation. The tilt deviation is set as ±0.01°. The offset deviation is set as ±1 micron. Figures 5, 6 and 7 show the simulation result for product A.

Simulation results show the following:

(1) Measurement errors caused by offset and tilt deviation increase when the distance between entrance slit and code dial decreases or the incident angle increases.
(2) Offset deviation causes more significant measurement errors. For product A, the measurement error will exceed 0.014° when *Δd* reaches 1 μm, as shown in Figure 5. Therefore, the measurement error caused by offset deviation should not be ignored.
(3) Tilt deviation has relatively less impact on the measurement errors. For product A, the measurement error is less than 0.005° at *Δφ* = 0.01°, as shown in Figure 6. For product B, the measurement error is much less. Meanwhile, the measurement error caused by *Δφ* varies with a period of 1° when the measurement angle is increased at 0.5° intervals in the simulation, as shown in Figure 7.

### 3.3. Algorithm Approximation Error in Signal Processing for Fine-Code

The encoded code dial includes coarse-code rows and fine-code rows. The final output angle is the sum of coarse-code output and fine-code output. The resolution of the ESS depends on the amount of code rows. The more the amount of code rows, the higher the resolution, but the highest resolution of coarse-code rows is only about 0.5°, which is limited by the divergence angle of the sun (0.53°). To improve the resolution further, fine-code rows is added to the code dial. Fine-code rows are not designed by dividing the coarse-code rows into smaller granularity, but adopting the principal that the output signal from the fine-code rows is a specific function of the incident angle *α*. The output angle of the fine-code can be derived by further processing the output of all fine-code rows. Typically, there are seven coarse-code rows and four fine-code rows. The four fine-code rows have the same pattern, while there is a phase difference of *θ*_0_/4 between two adjacent rows. *θ*_0_ is the period of fine-code, which is usually designed as 2°. For coarse-code output, an accuracy of 0.5° can be guaranteed. So the signal processing error of fine-code is the primary contributor to the algorithm error of ESS. Ideally, the output current of quad fine-code rows (F1∼F4) can be expressed as:

(1) $$\{\begin{array}{l} \text{F}1=\frac{a_{0}}{2}\left(1-\cos\left(\frac{2\pi }{\theta_{0}}\alpha \right)\right)\\ \text{F}2=\frac{a_{0}}{2}\left(1-\sin\left(\frac{2\pi }{\theta_{0}}\alpha \right)\right)\\ \text{F}3=\frac{a_{0}}{2}\left(1+\cos\left(\frac{2\pi }{\theta_{0}}\alpha \right)\right)\\ \text{F}4=\frac{a_{0}}{2}\left(1+\sin\left(\frac{2\pi }{\theta_{0}}\alpha \right)\right) \end{array}$$

where, *θ*_0_ is period of the fine-code rows; *a*_0_ is the amplitude of the fine-code output current; *α* is the measured angle (*i.e*., the incident angle of sunlight).

However, the actual output current of the quad fine-code rows does not follow the above formulas exactly [7]. Instead, they are periodic function similar to sine or cosine. It is well known that any periodic function can be expressed by Fourier series, so the actual output current of the quad fine-code rows can be expressed by Fourier series as:

(2) $$\{\begin{array}{l} \text{F}1=\frac{a_{0}}{2}-\sum_{n=1}^{\infty }a_{n}\cos(n\frac{2\pi }{\theta_{0}}\alpha )\\ \text{F}2=\frac{a_{0}}{2}-\sum_{n=1}^{\infty }a_{n}\sin(n\frac{2\pi }{\theta_{0}}\alpha )\\ \text{F}3=\frac{a_{0}}{2}+\sum_{n=1}^{\infty }a_{n}\cos(n\frac{2\pi }{\theta_{0}}\alpha )\\ \text{F}4=\frac{a_{0}}{2}+\sum_{n=1}^{\infty }a_{n}\sin(n\frac{2\pi }{\theta_{0}}\alpha ) \end{array}$$

where, *a*_0_ is the DC component, and *a*_n_ is the amplitude of harmonic component. It can be seen that the ideal output current (Equation (1)) includes only fundamental frequency and DC component of the actual one showed in Equation (2).

**Figure 8.**
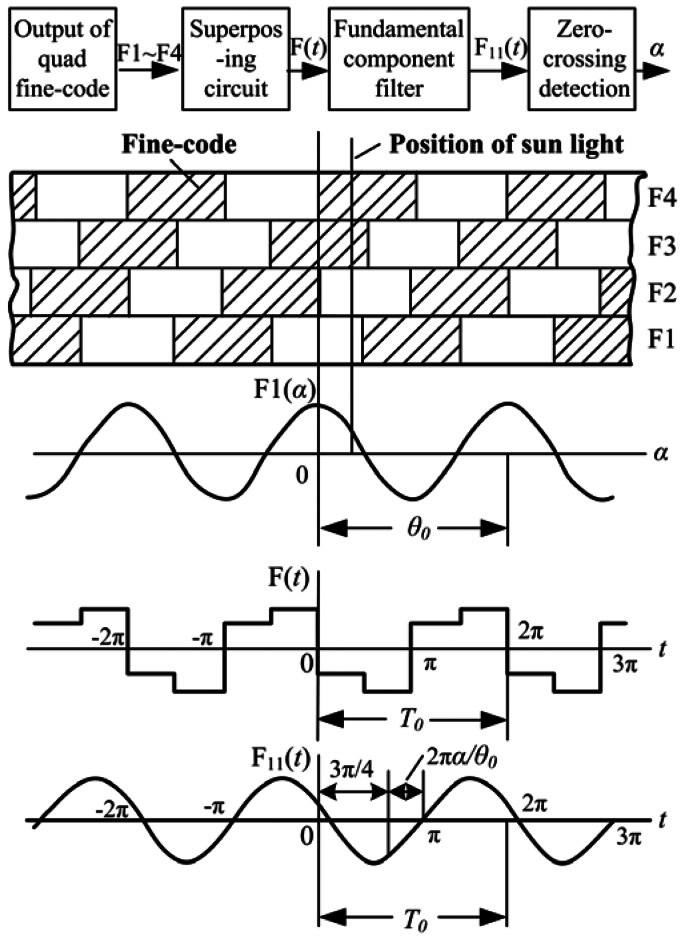
Working principle of fine-code processing circuit.

The fine-code signal processing circuit is based on a four-quadrant chopper. The working principle is shown in Figure 8. First, the fundamental components from the four fine-code rows are added together to get a superposed signal (designated as *F*(*t*)). Then, the harmonic component in the superposed signal *F*(*t*) is eliminated by a filter to get the fundamental component. Finally, an output which is related with the measurement angle *α* can be achieved by zero crossing detection.

If the output current of the quad fine-code rows is ideal, as shown in Equation (1), the fundamental component F_11_(*t*) of superposed signal F(*t*) should be in the following form:

(3) $${\text{F}}_{11}(t)=\frac{2\sqrt{2}}{\pi }a_{1}\sin(\omega t-\frac{3\pi }{4}-\frac{2\pi }{\theta_{0}}\alpha )$$

The relation between the zero-crossing phase *ωt*_0_ of the F_11_(*t*) and measurement angle *α* can be deduced by solving the equation of F_11_(*t*) = 0:

(4) $$\omega t_{0}=\frac{2\pi }{\theta_{0}}\alpha +\frac{3\pi }{4}$$

It can be seen that *ωt*_0_ is proportional to *α*, and there is a fixed phase difference of 3π/4. The phase difference can be compensated by a properly configured gate circuit. The proportional relationship between *ωt*_0_ and *α* is the theoretical base for the fine-code signal processing circuit.

The analysis above is based on the assumption that the output current of the quad fine-code rows is ideal, but from the actual output current shown in Equation (2), it is known that besides the fundamental component and the DC component, there are harmonic components. Among these harmonic components, even harmonics that have nothing to do with the measurement angle, while odd harmonics can bring errors to the measurement. Especially, the third harmonic can bring about a significant error because its amplitude is highest among the odd harmonics. Taking the third harmonic component into consideration, the corresponding fundamental component of superposed signal F(*t*) becomes:

(5) $${\text{F}}_{11}(t)=\frac{2\sqrt{2}}{\pi }a_{1}\sin\left(\omega t-\frac{3\pi }{4}-\frac{2\pi }{\theta_{0}}\alpha \right)+\frac{2\sqrt{2}}{\pi }a_{3}\sin\left(\omega t-\frac{3\pi }{4}-\frac{2\pi }{\theta_{0}}3\alpha \right)$$

where *a*_1_ is amplitude of fundamental component, and *a*_3_ is amplitude of third harmonic component. During the zero-crossing detection process, there is F_11_(*t*) = 0. That is:

(6) $$\frac{2\sqrt{2}}{\pi }a_{1}\sin\left(\omega t-\frac{3\pi }{4}-\frac{2\pi }{\theta_{0}}\alpha \right)+\frac{2\sqrt{2}}{\pi }a_{3}\sin\left(\omega t-\frac{3\pi }{4}-\frac{2\pi }{\theta_{0}}3\alpha \right)=0$$

Compared with Equation (3), the fine-code algorithm error *ε* caused by the third harmonic can be deduced and expressed as:

(7) $$\varepsilon =tg^{-1}\left[-\frac{\frac{a_{3}}{a_{1}}\sin(\frac{2\pi }{\theta_{0}}4\alpha )}{1+\frac{a_{3}}{a_{1}}\cos(\frac{2\pi }{\theta_{0}}4\alpha )}\right]$$

In actual design, the fundamental component is always much larger than the harmonic components. That is *a*_3_/*a*_1_ ≪ 1, so the equation above can be simplified to:

(8) $$\varepsilon \approx -\frac{a_{3}}{a_{1}}\sin(\frac{2\pi }{\theta_{0}}4\alpha )$$

### 3.4. Errors Introduced By the Etching Process and Non-Uniform Response of Photocell

Manufacturing errors in the etching process will result in amplitude and phase errors of the output current of fine-code rows. The non-uniform response of photocells will cause amplitude errors in the output current of fine-code rows [5]. Generally, these errors are less than ±1% on the average.

### 3.5. Classification of ESS Errors

From the analysis above, it can be seen that errors in ESS can be classified into three categories:

(1) Structural errors: these include distance deviations between entrance slit and code dial and offset and tilt deviations between the entrance slit and center line of the code dial. These errors belong to the system error class and are invariable. A model can be established to compensate them.
(2) Algorithm approximation errors of the fine-code: the errors are invariable too. A model can be deduced to compensate for them.
(3) Errors introduced by the etching process and non-uniform response of the photocell: the impact of this kind of error is insignificant. Furthermore, to establish a simple model is not easy because they are random errors.

## 4. Error Compensation Model for ESS

Based on the result of error analysis, the following compensation model for ESS is established. According to the fine-code algorithm error in Equation (8) and the phase error of fine-code output current, the fine-code algorithm error can be fitted by a sine function with parameters *k* and *t* as follows:

(9) $${\bar{\alpha }}_{2}=\alpha_{2}+k\sin(4\pi \alpha +t)$$

where *α_2_* is fine-code output angle, and *α̅_2_* is fine-code output angle after compensation. *α* is the incident angle of sunlight, *k* is the amplitude of the fine-code error, and *t* is the phase of the fine-code error.

As analyzed in Sections 3.1 and 3.2, structural errors mainly come from deviations in manufacturing and assembly. Structural errors can be deduced and calculated following the process of imaging (*i.e*., the process of coordinate transformation from the sunlight incident plane to the angle output in the code dial reference frame).

Suppose the sunlight incident angle is *α*. Ideally, the sunlight incidence plane can be expressed as:

(10) y=tan(α)⋅z

where *y* and *z* are coordinate values of the incident plane.

Taking structural errors (mentioned in Section 3.1 and 3.2) into consideration, the coordinate of the intersection of the incident plane and Y axis can be expressed as:

(11) $$y\prime =\frac{H\cdot a\cdot \tan(\alpha )+b\cdot H}{c\cdot \tan(\alpha )+d}$$

where, *a*, *b*, *c*, *d* are conversion coefficients between the sunlight incidence plane and the code dial reference frame. Among them, *a* and *d* are the parameters associated with the distance between the entrance slit and code dial, while *b* and *c* are the parameters associated with the offset and tilt between entrance slit and the center line of the code dial.

The corresponding incident angle in the code dial reference frame can be expressed as:

(12) $$\alpha_{d}=\text{atan}(\frac{y\prime }{H})=\text{atan}\left(\frac{a\cdot \tan(\alpha )+b}{c\cdot \tan(\alpha )+d}\right)$$

where, *α_d_* is the incident angle transferred to the code dial reference frame. After conversion by coarse-code rows and fine-codes rows, the coarse-code output *α*_1_ and fine-code output *α*_2_ are obtained.

According to the fine-code algorithm error shown in Equation (9) and the structural error shown in Equation (12), the error compensation model for ESS can be expressed as:

(13) $$\alpha_{\text{comp}}=\text{atan}\left(\frac{b-d\cdot \tan(\alpha_{s})}{c\cdot \tan(\alpha_{s})-a}\right)$$

where, *α_s_* = *α*_1_ + *α̅*_2_. *α*_1_ is the output angle of coarse-code. *α̅*_2_ is output angle of fine-code after compensation. *α_comp_* is the final output angle of ESS after compensation.

## 5. Calibration of Parameters of the Error Compensation Model

**Figure 9.**
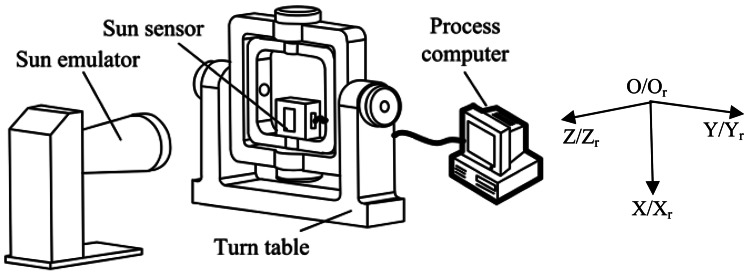
Setup of calibration system.

After the error compensation model is established, the model parameters can be calibrated with the equipment shown in Figure 9. The ESS is mounted on the inner-frame of a turntable, and sunlight is provided by a sun emulator. Rotating the inner-frame and external frame of the turn-table by angles *α* and *β* respectively have the same effect as sunlight projecting onto the ESS with an incident angle α and incoming angle *β*. Here, the incoming angle is the intersection angle between the sunlight and the YZ plane of the ESS. Measurement results will be sent to a computer for processing. The turntable will be rotated to m sets of predefined calibration angles. These m sets of angles (*α_i = 1∼m_*, *β_i = 1∼m_*) and its corresponding ESS outputs *α_ci = 1∼m_* form an array of calibration data [8,9].

For convenience, the turntable reference frame Or-XrYrZr is defined in the following way: when the turntable is at the zero position, the inner-frame rotation axis is Xr, and the external-frame rotation axis is Yr. Axis Zr is defined by the right-hand rule. The ESS reference frame O-XYZ is defined in the following way: the center of the code dial is O, axis X is perpendicular to the code row direction; axis Y is parallel to the code row direction; and axis Z is defined by the right-hand rule.

Suppose the installation deviation is zero in the calibration system, that is: (a) verticality deviation between the optical axis of the solar simulator and the turntable reference frame Or-XrYrZr is zero; (b) deviation between O-XYZ and Or-XrYrZr is zero. Then, the incoming angle *β* has no relation with the incident angle *α* and can have any value. A series of predetermined calibration angles can be generated merely by rotating the inner frame at certain intervals, but in reality, installation deviation is unavoidable and incoming angle *β* and incident angle *α* are related. The calibration data has to be acquired for different incoming angles in order to guarantee the accuracy of parameter calibration. Furthermore, all calibration data should be acquired at the same initial zero position of the turntable [10,11].

**Figure 10.**
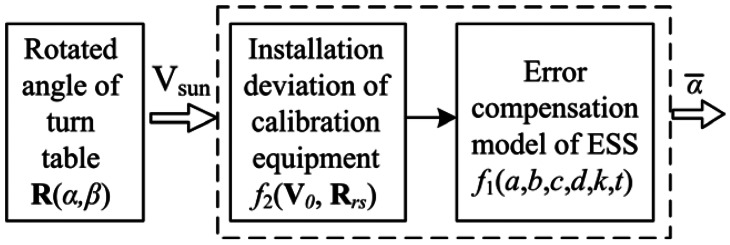
Parameters in the ESS calibration model.

On the other hand, because of the installation deviation of calibration equipment, the calibration accuracy for parameters will be decreased if the calibration is directly according to the ESS error compensation model. Those installation deviations get involved inevitably during the process of parameter calibration. As a result, these external installation errors have to be taken into account to establish the integrated ESS calibration model as shown in Figure 10.

The accurate calibration model for ESS can be expressed as a function containing installation deviation parameters **V**_0_ and **R**_rs_ as well as error compensation model parameters *a*, *b*, *c*, *d*, *k* and *t*:

(14) $$\begin{array}{ll} \bar{\alpha } & =F({\text{V}}_{0},{\text{R}}_{rs},a,b,c,d,k,t,\alpha ,\beta )\\ & =f_{1}(a,b,c,d,k,t)\times \text{R}(\alpha ,\beta )\times f_{2}({\text{R}}_{rs},{\text{V}}_{0})\\ & =f_{1}(a,b,c,d,k,t)\times \text{R}(\alpha ,\beta )\times {\text{R}}_{rs}(r_{1}\sim r_{9})\times {\text{V}}_{0}(e_{1}\sim e_{3})\\ & =f_{1}(a,b,c,d,k,t)\times \left[\begin{array}{ccc} 1 & 0 & 0\\ 0 & \cos\alpha & \sin\alpha \\ 0 & -\sin\alpha & \cos\alpha \end{array}\right]\times \left[\begin{array}{ccc} \cos\beta & 0 & -\sin\beta \\ 0 & 1 & 0\\ \sin\beta & 0 & \cos\beta \end{array}\right]\times \left[\begin{array}{ccc} r_{1} & r_{2} & r_{3}\\ r_{4} & r_{5} & r_{6}\\ r_{7} & r_{8} & r_{9} \end{array}\right]\times \left[\begin{array}{c} e_{1}\\ e_{2}\\ e_{3} \end{array}\right] \end{array}$$

In Equation (14), **V***_0_* is the verticality deviation vector between sunlight and the turntable reference frame, and is represented by a 3 × 1 vector. **R***_rs_* is the installation deviation matrix between the ESS and the turntable, and can be described by a 3 × 3 conversion matrix between the ESS and the turntable reference frame. Terms *α* and *β* are the known calibration angles (*i.e*., rotation angles of the inner and external frames of the turntable, respectively); **R**(*α*, *β*) is the corresponding rotation matrix of the turn-table. *f*_1_(*a*, *b*, *c*, *d*, *k*, *t*) is the ESS error compensation function shown in Equations (9) and (13). *f*_2_(**R***_rs_*, **V**_0_) is the function of installation deviation and equal to the product of **R***_rs_* and **V**_0_.

It can be seen in Equation (14) that there are eighteen parameters that need to be optimized for solutions, namely, (*a*, *b*, *c*, *d*, *k*, *t*, *e_1_*, *e_2_*, *e_3_*) and (*r_1_*–*r_9_*). Among them, (*a*, *b*, *c*, *d*, *k*, *t*) are the error compensation parameters of the ESS itself, which are defined as internal parameters of the ESS. (*e_1_*, *e_2_*, *e_3_*) and (*r_1_*–*r_9_*) are installation deviation parameters between the ESS and the calibration equipment, which are defined as external parameters of the ESS. *α̅* is the estimated value of ESS output angle calculated by the calibration model.

The calibration data are substituted into the calibration model shown in Equation (14). All parameters (including installation deviation parameters and ESS error compensation parameters) can be optimized by the Least Squares method. The specific optimization procedure is as follows:

Rotated angles of the turntable (*α_i = 1∼m_*, *β_i = 1∼m_*) are substituted into the calibration model shown in Equation (14) to get the estimated value *α̅_i=_*_1∼_*_m_* of the ESS output angle. Combined with the actual value *α_ci = 1∼m_*, of the ESS output angles, a system of equations containing m equations is formed, as shown in Equation (15).

(15) $$\{\begin{array}{l} \Delta \alpha_{1}={\bar{\alpha }}_{1}-\alpha_{c1}\\ \vdots \\ \Delta \alpha_{m}={\bar{\alpha }}_{m}-\alpha_{cm} \end{array}$$

It can be seen that the Equation (15) is an over-determined equation system involving eighteen parameters. The equation can only be solved by optimization. In the paper, s Nonlinear Least Squares iteration algorithm is used to solve the parameters. The detailed procedure is as follows:

Equation (15) can be expressed in the following vector form:

(16) $$\Delta \alpha =\bar{\alpha }-\alpha_{\text{c}}=F({\text{V}}_{0},{\text{R}}_{rs},a,b,\text{c},d,k,t,\alpha ,\beta )-\alpha_{c}\approx \text{A}\Delta \text{P}$$

where **Δα** is a vector made up of (Δ*α*_1_⋯Δ*α*_m_). **ΔP** is the deviation vector of the optimized parameters mentioned above. **A** is a partial derivative vector. Its expression is:

(17) $$\text{A}=\left[\begin{array}{llllllllllll} \frac{\partial F}{\partial a} & \frac{\partial F}{\partial b} & \frac{\partial F}{\partial c} & \frac{\partial F}{\partial d} & \frac{\partial F}{\partial k} & \frac{\partial F}{\partial t} & \frac{\partial F}{\partial e_{1}} & \frac{\partial F}{\partial e_{2}} & \frac{\partial F}{\partial e_{3}} & \frac{\partial F}{\partial r_{1}} & \cdots & \frac{\partial F}{\partial r_{9}} \end{array}\right]$$

Based on the nonlinear least squares iteration algorithm, the following iteration equation can be established:

(18) $$\Delta {\text{P}}^{k+1}=\Delta {\text{P}}^{k}-{\left({\text{A}}_{k}^{T}{\text{A}}_{k}\right)}^{-1}{\text{A}}_{k}^{T}\Delta \alpha^{K}$$

where *k* is the iteration index. The initial values of *a*, *b*, *c*, *d*, *k*, *t* for the iteration algorithm are set as *a* = *d* = H, *b* = *c* = 0, *k* = 0.03, *t* = 0 respectively. The initial value of **R**_rs_(*r*_1_∼*r*_9_) is set as the identity matrix and **V**_0_(*e*_1_∼*e*_3_) is set as [0,0,1]^T^. Usually, the estimated parameters in Equation (18) can converge to a stable value after five to ten instances of iteration.

## 6. Experimental Results

Calibration and accuracy verification experiments are carried out on a large number of products. They are tested within different ranges of incident angles, tested at different sample intervals of incident angle and different installation deviations of the calibration system. The experimental results are shown in Tables 1, 2 and 3. Table 1 shows the statistical results for twenty products randomly selected, which are tested in different ranges of the incident angle.

**Figure 11.**
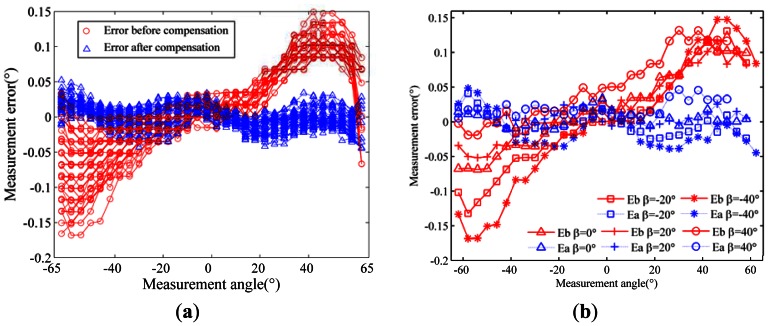
Measurement errors for one product of type B before and after compensation. ((**a**) all curves for different *β* values (**b**) a few representative curves selected from (a)).

Table 2 shows the results for two products at different sample intervals of the incident angle. Table 3 shows the error compensation parameters calibrated for one product under different installation deviation between ESS and turntable. Figure 11 shows a typical result for one product of type B, whose RMS error is 0.0635° before compensation and 0.0157° after compensation, and whose calibrated parameters are shown in Table 4. Measurement errors before and after compensation are shown in Figure 11(a), in which each curve represents one set of measurement errors for different incoming angles *β.* For clarity, a few representative curves are selected from (a) and plotted in Figure 11(b), in which “Eb” represents “error before compensation” and “Ea” represents “error after compensation”.

Experimental results show the following:

(1) From Table 1, it can be seen that accuracy improvement is greater within 30° ∼ 60° than that within ±30°, and the accuracy improvement for product A is greater than that for Product B. This is because measurement errors caused by structural errors increase when H decreases or the incident angle increases as analyzed in Section 3.
(2) From Table 2, it can be seen that the compensation effect with a 0.25° sampling interval is better than that with a 1°sampling interval, but the improvement is limited. It agrees with our theoretical analysis, and here are reasons for that:
  (a) Because the fine-code algorithm compensation equation is a sine function with a period of 0.5°, the compensation effect is improved when the calibration data is sampled at an interval shorter than the period of 0.5°.
  (b) The solved parameter k is generally less than 0.03, which indicates that the impact of the fine-code algorithm error is relatively minor, so there is a limit on the improvement.
(3) From Table 2, it can be seen that the error compensation parameters calibrated under different installation deviations are almost identical, which indicates that the calibration model and method proposed in the paper effectively avoid the impact of installation deviations of the calibration equipment.
(4) From Table 1, it can also be seen that after compensation, the ESS measurement accuracy is improved by three times on average within ±60° of the incidence angle.

## 7. Conclusion

Various kinds of error in ESS are analyzed in this paper. The result shows that the structural errors which occurred during the manufacturing and assembly processes, together with the algorithm approximation errors of the fine-code, are the major factors which limit the measurement accuracy of ESS units. Based on the analyzed results, an error compensation model is established to compensate the errors mentioned above.

In addition, an accurate parameter calibration method is also proposed. To avoid the impact of installation deviations of the calibration system on the accuracy of parameter calibration, an accurate calibration model of ESS is established which takes the installation deviation of calibration equipment into consideration. The experimental results show that after compensation, the ESS measurement accuracy is improved by three times on average within ±60° of the incidence angle. The technology described in the paper has been successfully applied to sun sensor products of a certain research institute in China.

## Figures and Tables

**Table 1.** Statistical results for twenty products of type A and B.

**Product Type**	**Range of Incident Angle**	**RMS Error after Compensation**		**Times of Accuracy Improvement**		

		**Max**	**Min**	**Mean**	**Max**	**Min**

Type A (H = 4.124 mm)	±30°	0.0113°	0.0071°	4.76	6.04	3.25
	±30°∼±60°	0.0229°	0.0099°	5.526	6.8	3.96
	±62°	0.0245°	0.0093°	3.94	5.17	2.7

Type B (H = 6.584 mm)	±30°	0.010°	0.005°	2.88	4.5	1.49
	±30°∼±60°	0.0143°	0.0073°	4.47	5.18	3.07
	±62°	0.157°	0.0056°	3.34	4.27	2.83

**Table 2.** Results for two products at different sample intervals.

**Product Type**	**Sample Interval**	**Range of Incident Angle**	**RMS Error before Compensation**	**RMS Error after Compensation**

Type A (H = 4.124 mm)	1°	±62°	0.0399°	0.0141°
	0.25°	±62°	0.0296°	0.0102°

Type B (H = 6.584 mm)	1°	±62°	0.020°	0.007°
	0.25°	±62°	0.0191°	0.0056°

**Table 3.** Model parameters calibrated under different installation deviation.

**Deviation Between O-XYZ and Or-XrYrZr**			**Error Compensation Parameters**					

Axis X	Axis Y	Axis Z	*a*	*b*	*c*	*d*	*k*	*t*
0	0	0	4.1289	−0.0003	−0.0003	4.1191	−0.0225	−0.7787
0	5′	38″	4.1292	−0.0001	−0.0002	4.1188	−0.0164	−0.8623
17″	1′34″	1′8″	4.1289	−0.0003	−0.0004	4.1191	−0.0177	−0.8005

**Table 4.** Model parameters calibrated for one product of type B.

**a**	**b**	**c**	**d**	**k**	**t**	**r_1_**	**r_2_**	**r_3_**
6.5912	−0.0005	−0.0006	6.5759	0.0357	2.4558	1.0000	0.0004	0.0014
r_4_	r_5_	r_6_	r_7_	r_8_	r_9_	e_1_	e_2_	e_3_
−0.0004	1.0000	−0.0003	−0.0014	0.0003	1.0000	−0.0066	0.0001	1.0000
